# Supplementary material for: Effect of Kinase Inhibiting RNase Attenuator (KIRA) Compounds on the Formation of Face-to-Face Dimers of Inositol-Requiring Enzyme 1: Insights from Computational Modeling
Source: Int J Mol Sci. 2019 Nov 6;20(22):5538. doi: 10.3390/ijms20225538 (PMC6887741; doi:10.3390/ijms20225538)
Supplement: Supplementary file 1 [file ijms-20-05538-s001.pdf]

Supplementary Information

Effect of Kinase Inhibiting RNase Attenuator (KIRA) Compounds on the Formation of Face-to-Face Dimers of Inositol-Requiring Enzyme 1: Insights from Computational Modeling

Antonio Carlesso [1], Chetan Chintha [2], Adrienne M. Gorman [2], Afshin Samali [2] and Leif A. Eriksson [1,*]

[1] Department of Chemistry and Molecular Biology, University of Gothenburg, 405 30 Göteborg, Sweden;

[2] Apoptosis Research Centre, National University of Ireland Galway, H91 TK33, Galway, Ireland

* Correspondence: leif.eriksson@chem.gu.se; Tel.: +46 317869117

Table S1. RMSD[a] for the 5 top-scored docked poses generated using five different protein–protein docking approaches to reproduce the known IRE1 dimer complexes.

	**Face-to-face dimer (PDB code: 3P23)**	**Back-to-back dimer (PDB code: 4YZC)**
**SwarmDock**	1.39, 27.47, 12.94, 16.43, 1,42	3.56, 31.58, 23.99, 20.23, 34.32
**ZDOCK**	12.48, 14.59, 0.97, 16.24, 3.65	3.32, 23.70, 31.57, 33.97, 13.34
**HsymDock**	3.12, 33.20, 39.72, 38.63, 33.62	13.25, 12.84, 30.68, 28.96, 35.72
**PatchDock**	24.33, 30.03, 25.45, 28.00, 32,59	29.49, 21.59, 28.19, 25.35, 21.79
**ClusPro**	3.58, 11.91, 22.40, 17.65, 29.22	31.01, 33.02, 30.02, 34.60, 30.10

[a]Root-mean-square deviation (RMSD) is calculated for Cα atoms by superimposing the five top-scored docked poses generated by the programs, with the crystallographic structures.

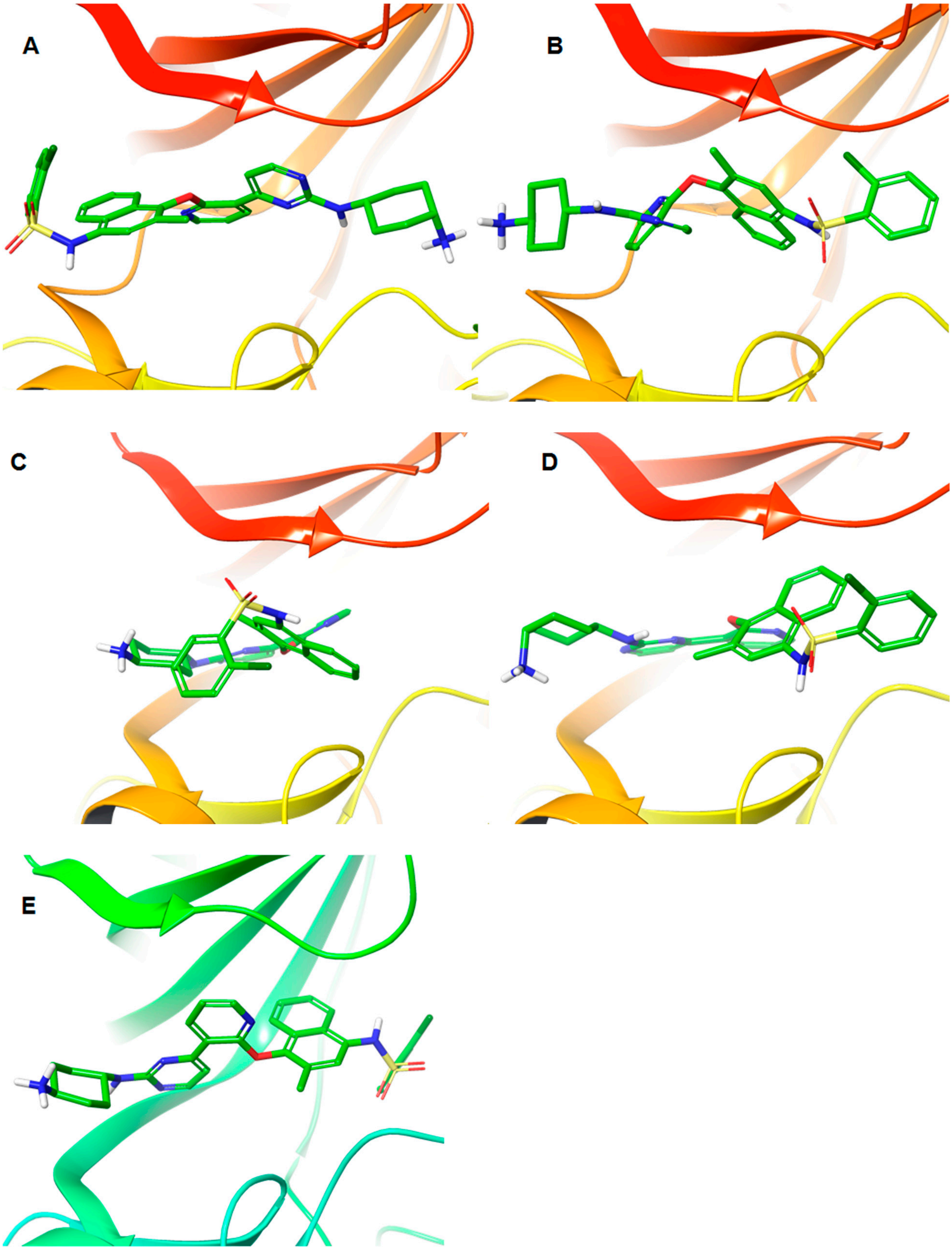

Figure S1. Representation of the KIRA docking pose in (**A**) Chain A of 3P23 PDB code, (**B**) Chain B of 3P23 PDB code, (**C**) Chain A of 4YZC PDB code, (**D**) Chain B of 4YZC PDB code. The crystallographic pose of KIRA in the 4U6R PDB structure is shown in panel **E**.

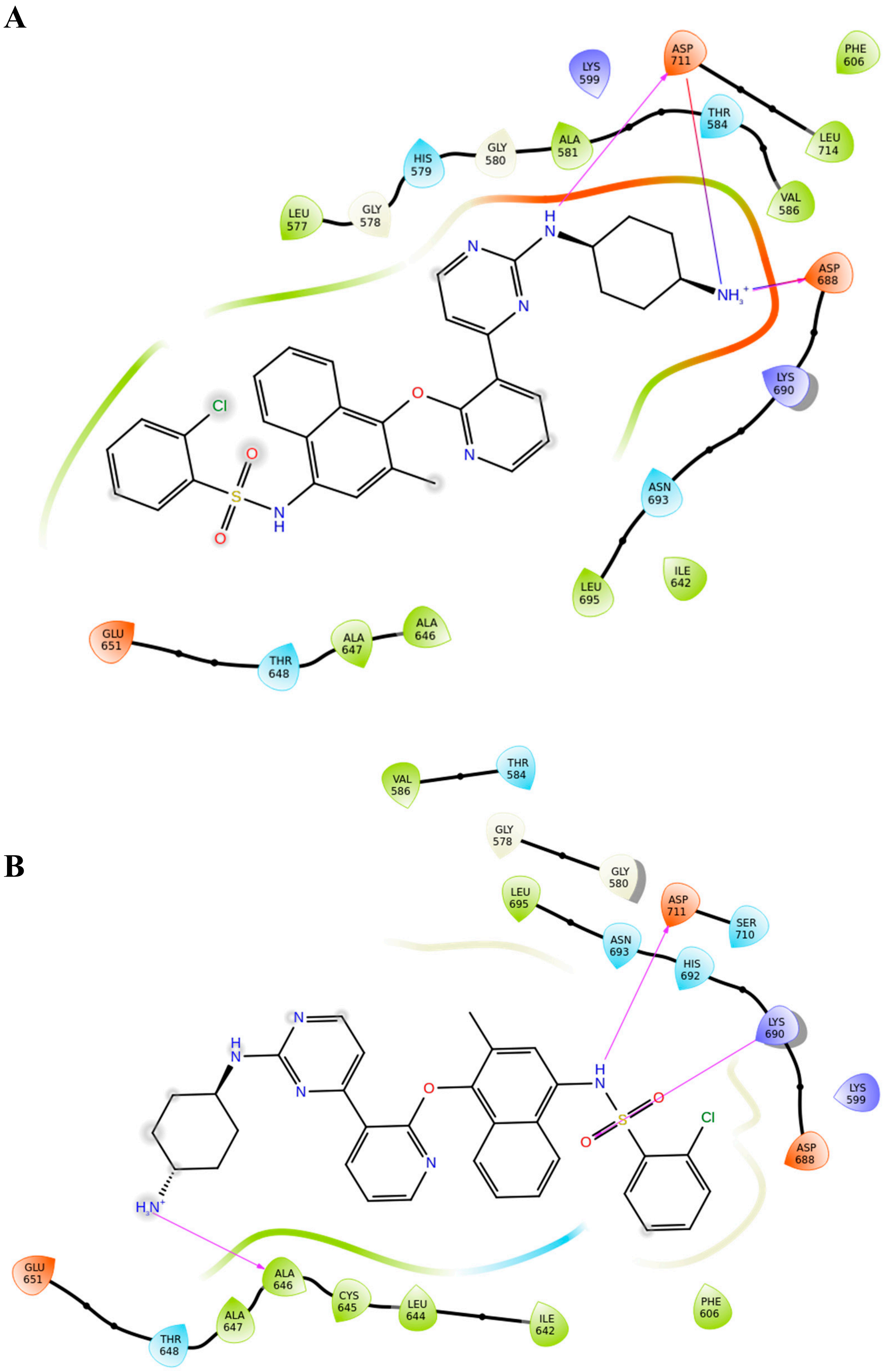
A
ASP 711
PHE 606
LYS 599
THR 584
LEU 714
ALA 581
GLY 580
HIS 579
VAL 586
GLY 578
LEU 577
ASP 688
LYS 690
ASN 693
ILE 642
LEU 695
GLU 651
THR 648
ALA 647
ALA 646
Cl
O
S
N
H
NH3+
B
VAL 586
THR 584
GLY 578
GLY 580
LEU 695
ASP 711
SER 710
ASN 693
HIS 692
LYS 690
LYS 599
ASP 688
GLU 651
ALA 646
CYS 645
LEU 644
ILE 642
ALA 647
THR 648
PHE 606
H3N+

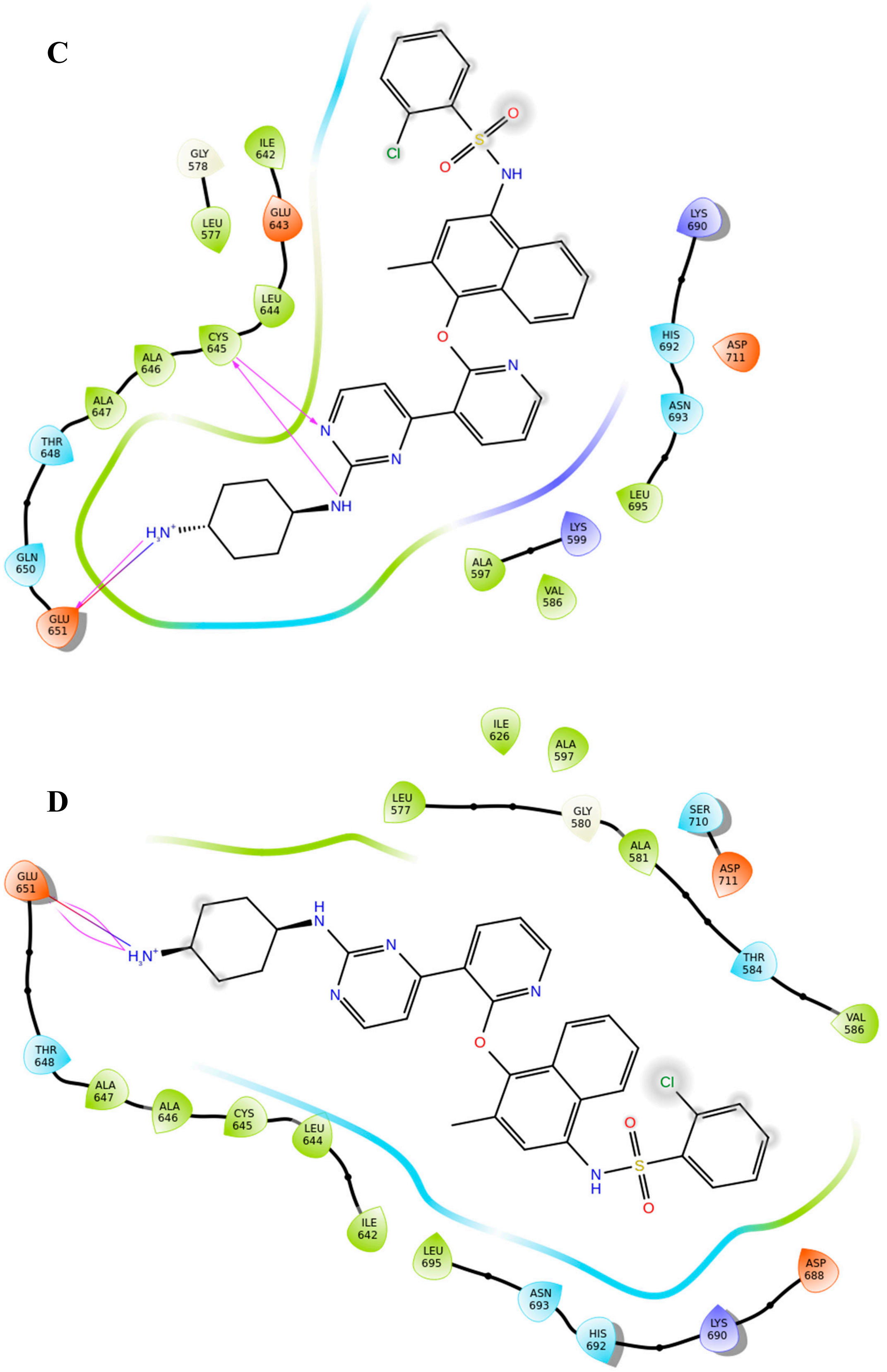

Figure S2. 2D representation diagrams of the KIRA binding modes in (**A**) Chain A of 3P23 PDB code, (**B**) Chain B of 3P23 PDB code, (**C**) Chain A of 4YZC PDB code, and (**D**) Chain B of 4YZC PDB code. Interactions between the IRE1 residues and the ligand are drawn as lines, colored by interaction type. Arrows indicate H-bonds, with the direction from donor to acceptor.

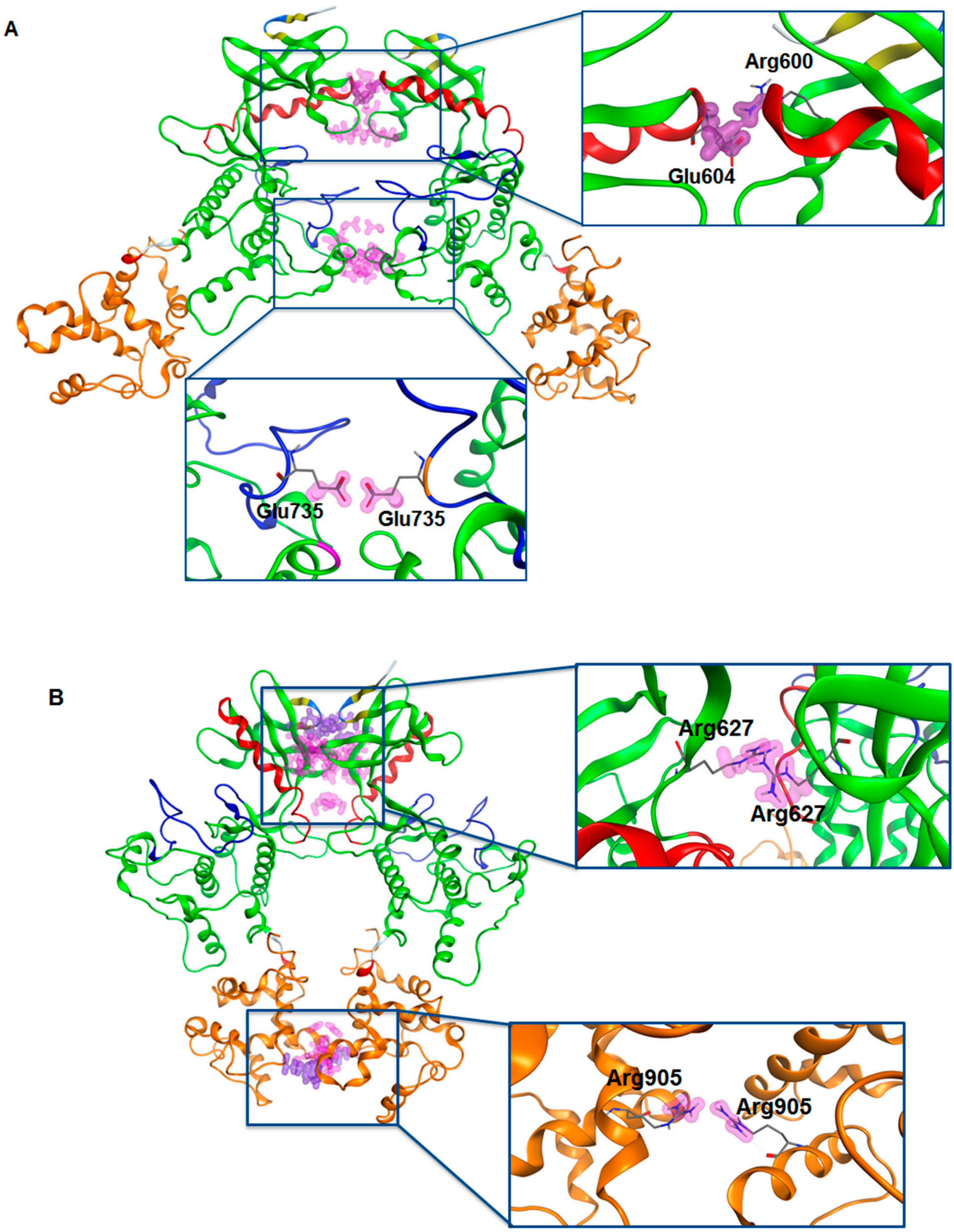

Figure S3. Ribbon diagram representing the structure of the KIRA-bound dimer forms obtained by superposition of the monomer of the 4U6R PDB structure on each monomer of the native crystallographic structures of the IRE1 in (A) face-to-face (PDB 3P23) and (B) back-to-back (PDB code: 4YZC) dimers. The kinase domain is shown in green (residues 571-832), the helix-αC in red (residues 603-623), the activation segment in blue (residues: 711-741), the RNase domain in orange (residues 837-963). The yellow segment represents the β-strand and the cyan the H-bonded turn that are not part of the kinase or RNase domain. Violet spheres = steric clashes.

A

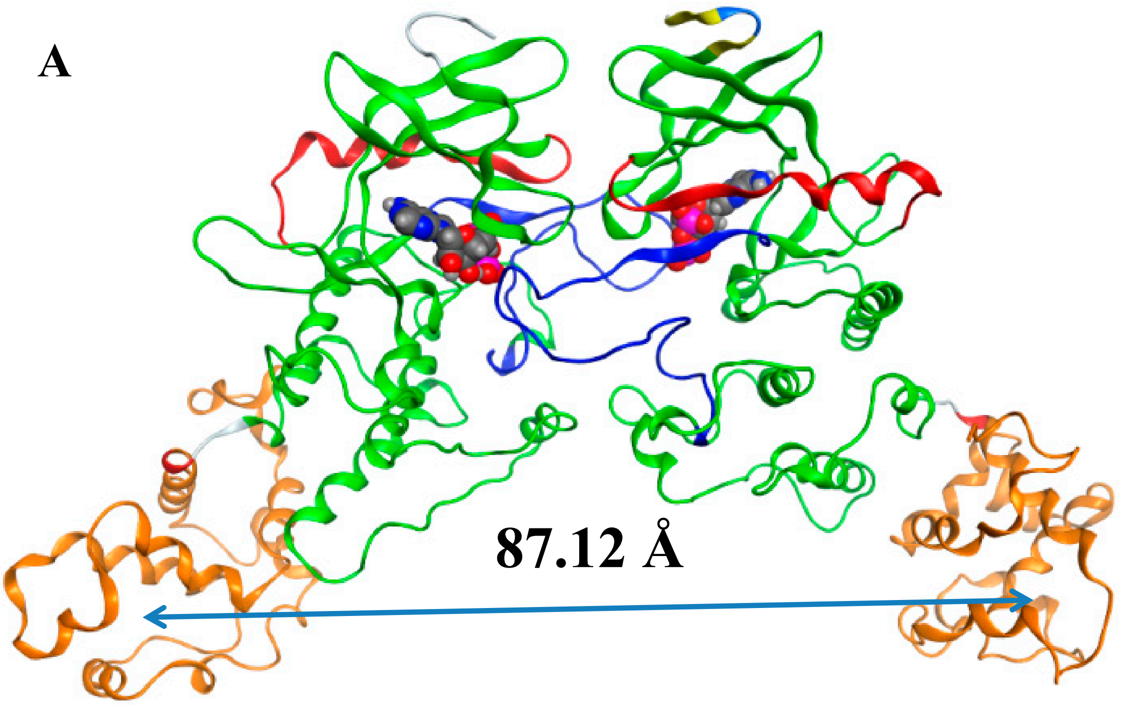

B

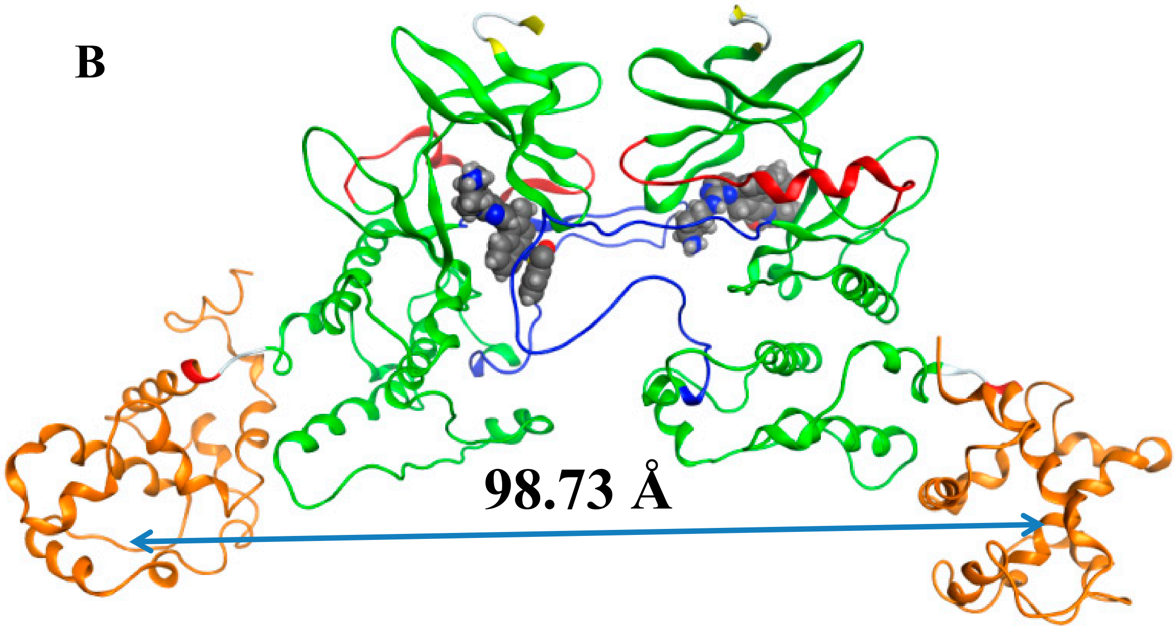

C

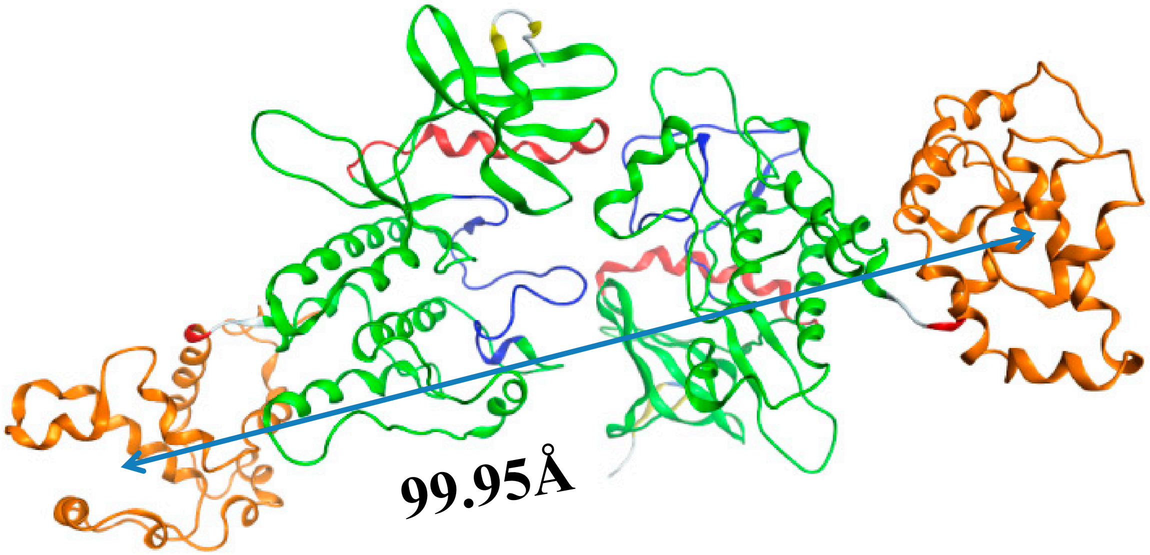

Figure S4. Comparison of the (**A**) native face-to-face crystal dimer structure (PDB code: 3P23) (point 'a' in Panel D of Figure 5), with the higher interface RMSD frame identified from three independent MD replicas for (**B**) KIRA docked in PDB 3P23 dimer (point 'b' in Panel E of Figure 5) and (**C**) protein-protein docked pose of PDB 4U6R in face-to-face dimer (point 'c' in Panel F of Figure 5). The distance between the RNase domain Center of Mass (COM) of dimer is shown. The kinase domain is shown in green (residues 571-832), the helix-αC in red (residues 603-623), the activation segment in blue (residues: 711-741), the RNase domain in orange (residues 837-963). The yellow segment represents the β-strand and the cyan the H-bonded turn that are not part of the kinase or RNase domain. ADP (**A**) and KIRA (**B**) highlighted in space-filling model to indicate the kinase binding site.

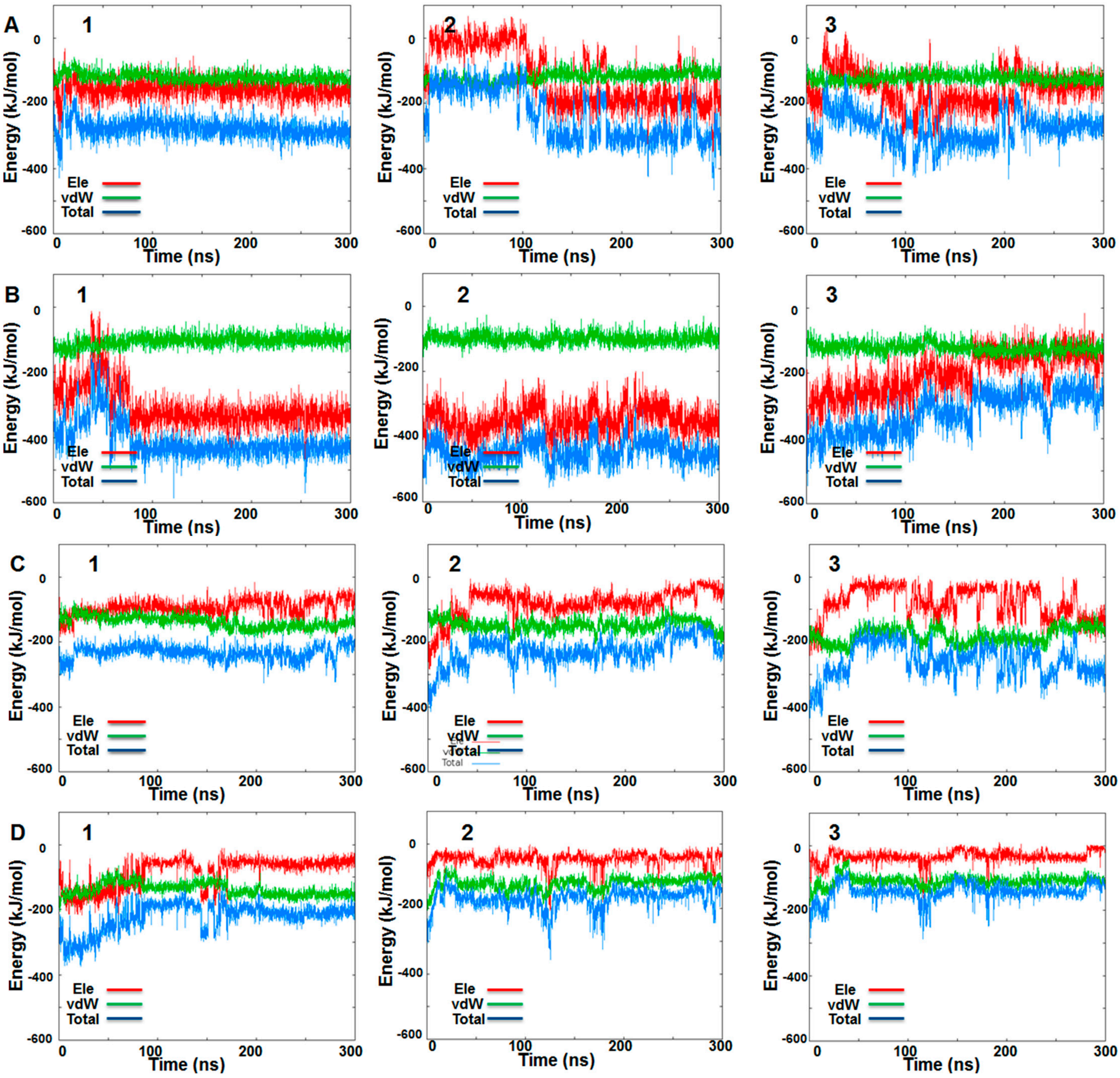

Figure S5. IRE1 face-to-face dimer MD simulations. Time-resolved interaction energy profiles for ADP during the three MD simulation replicas of the native face-to-face crystal dimer (PDB code: 3P23): (**A**) Chain A, (**B**) Chain B. Time-resolved interaction energy profiles during the three MD simulation replicas for KIRA docked in the native face-to-face crystal dimer (PDB code: 3P23): (**C**) Chain A and (**D**) Chain B.

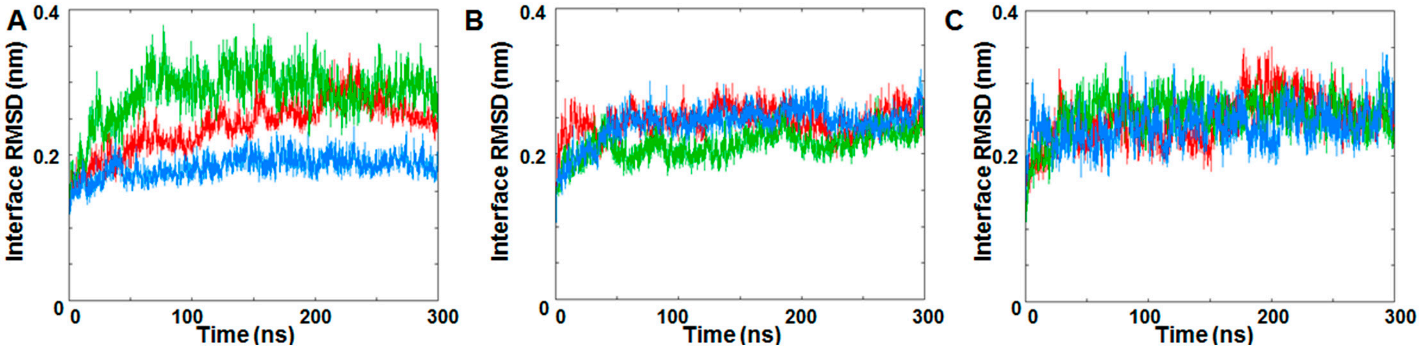

Figure S6. Interface RMSDs of IRE1 back-to-back dimer Cα atoms during the three MD simulation replicas of (**A**) native back-to-back crystal dimer structure (PDB code: 4YZC), (**B**) KIRA docked in PDB 4YZC dimer, and (**C**) protein-protein docked pose of PDB 4U6R in back-to-back dimer form. Replicates 1, 2, and 3 are represented in red, green and blue, respectively. .

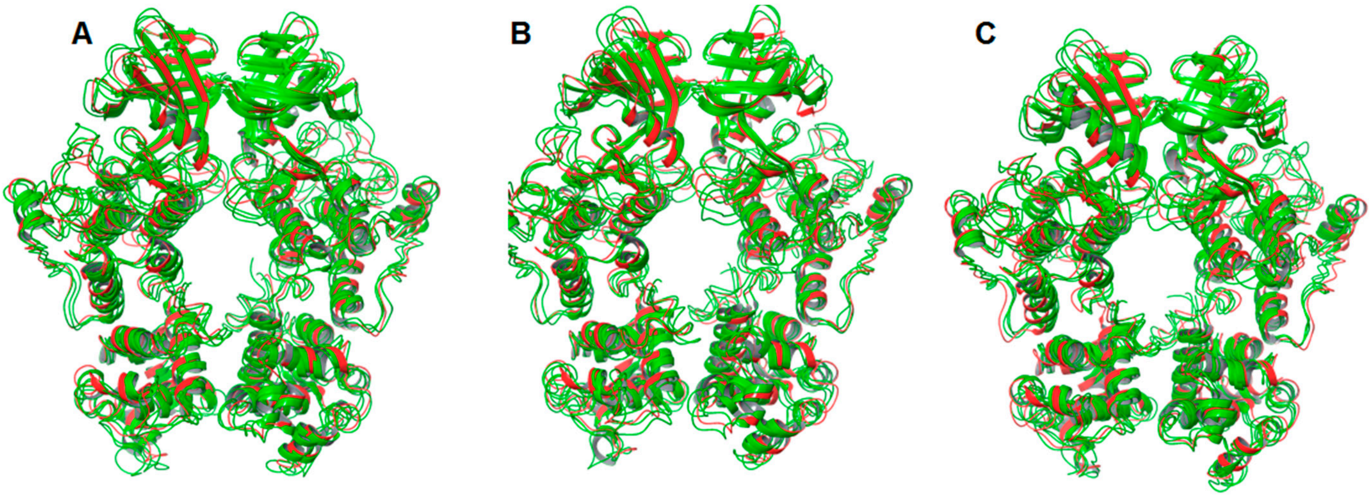

Figure S7. Superposition of the last frame of each individual MD simulation (green) of (**A**) native back-to-back dimer from crystal structure (PDB code: 4YZC), (**B**) KIRA docked in PDB 4YZC structure and (**C**) protein-protein docked pose of PDB 4U6R in back-to-back dimer, onto the native back-to-back crystallographic structure (PDB code: 4YZC) (red).

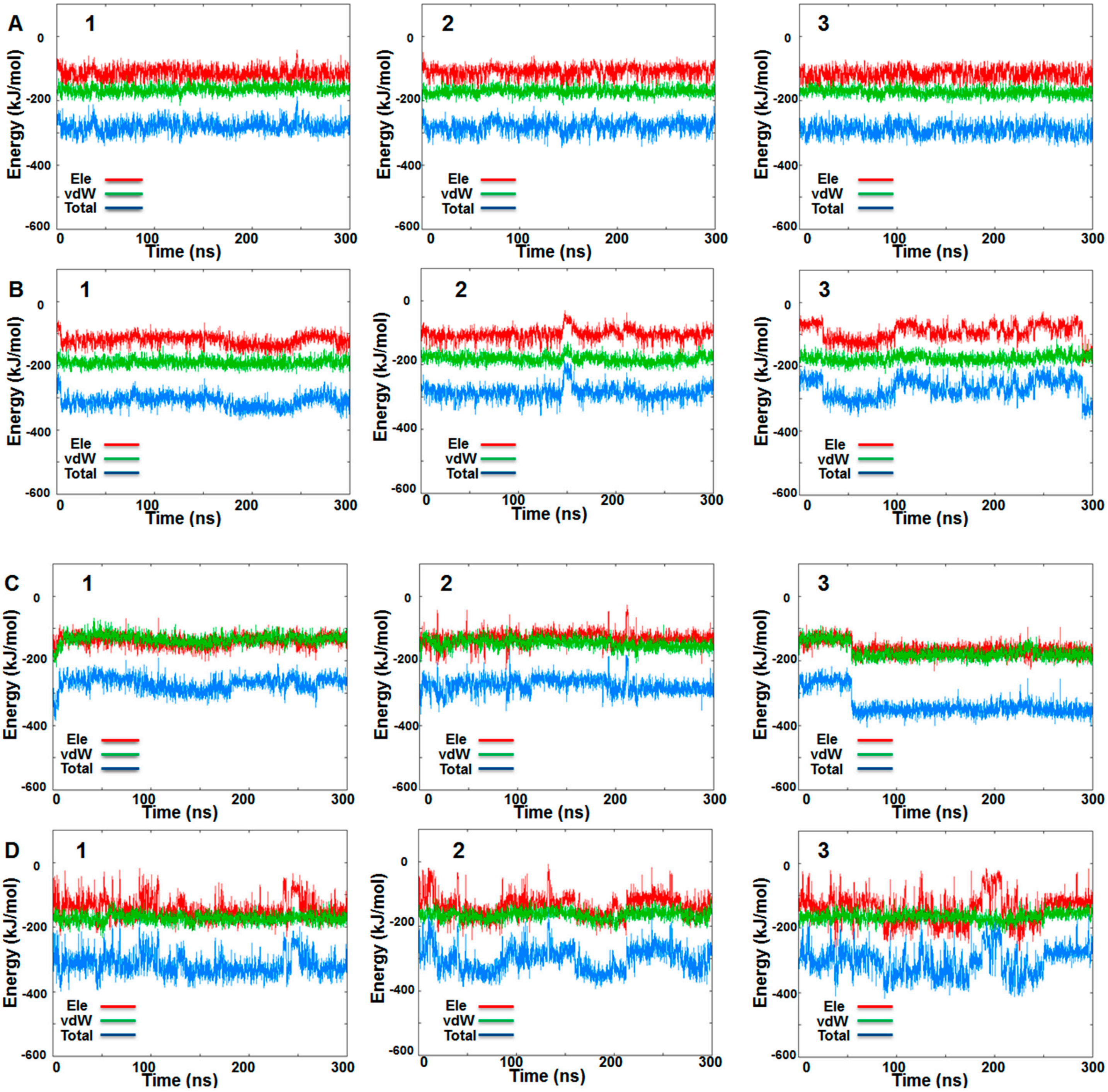

Figure S8. IRE1 back-to-back dimer MD simulations. Time-resolved interaction energy profiles for staurosporine during the three MD simulation replicas of the native back-to-back crystal dimer (PDB code: 4YZC): (**A**) Chain A, (**B**) Chain B. Time-resolved interaction energy profiles during the three MD simulation replicas for KIRA docked in the native back-to-back crystal dimer (PDB code: 4YZC): (**C**) Chain A and (**D**) Chain B.
